# Poor-Law Infirmaries and Voluntary Hospitals

**Published:** 1913-02-01

**Authors:** A. P. Hughes Gibb

**Affiliations:** Assistant Secretary of the Seamen's Hospital Society.


### POOR-LAW INFIRMARIES AND VOLUNTARY HOSPITALS.

### Co-operation in the Treatment of the Sick.

By A. P. HUGHES GIBB, Assistant Secretary of the Seamen's Hospital Society.

Voluntary hospitals and Poor-Law infirmaries both exist for the care and treatment of the sick poor, yet there is hardly a vestige of co-operation between them; these two great organisations carry on their work side by side without in most cases even the most formal communications passing between them. There appear to be no statistics available of the number of cases treated in Poor-Law infirmaries in London which on medical or surgical grounds would be equally suitable for a voluntary hospital; but as the London infirmaries contain nearly twice as many beds (18,248—1911) as the hospitals of the Metropolis (9,538—1910), the acute cases in each are probably about the same in number.

### The Almoner's Position.

It has frequently been suggested that there is a social line of demarcation between the persons making use of the Poor-Law infirmaries and those turning to hospitals for relief. It has been said that the object of the latter should be to prevent the poor from falling into destitution, and of the former to relieve those cases which have gone a stage further and are actually destitute. In fact, it certainly is not the general practice of hospitals to turn away applicants for relief on the ground of poverty alone: such an action would in most cases be inconsistent with the intentions of the founders and supporters of voluntary hospitals.

At present the main question which an almoner has to answer is: "Can the applicant for relief afford to pay for the particular treatment which his case requires?" Supposing the answer to be in the negative, on what grounds can an inquiry officer decide further whether the patient has reached such a stage of destitution as to be suitable only for relief by the Poor Law. The only distinction at present existing is largely one of chance. Persons already in receipt of out-door relief and in touch with the Poor-Law officers naturally tend to go to the infirmary in case of illness; but there is nothing to prevent their applying for and receiving relief from a voluntary hospital, and in many cases they do so.

On the other hand, the sickness of the breadwinner frequently produces immediate destitution, and the hospital can do no more to prevent this occurring than the Poor-Law infirmary; both do the same work by removing as soon as possible the cause of poverty—the cessation of the earnings of the breadwinner. The tramp, the wastrel, and the loafer, who scrape a precarious livelihood with the assistance of mistaken charity, will seek independently the infirmary or the hospital in time of illness, and will get relief in either.

### The Spheres of Hospital and Infirmary.

Thus I venture to maintain that there is at present practically no differentiation in principle or practice between the cases treated by the infirmary and those relieved by the voluntary hospital, and that any distinction made on the grounds of poverty or destitution cannot be generally applied because the financial position of the class dealt with varies with such great rapidity, and may be immediately depressed to the point of destitution by the illness of the breadwinner.

Both these great agencies for medical and surgical relief of the poor have their weak points, and it is possible that by a closer co-operation between them each might supply to some extent the weakness of the other. In seeking to mark out different spheres for the activity of each, it has all along been assumed that the line of demarcation should be social.

In the minds of investigators the taint of the Poor Law, a dark stigma, attaches to relief in an infirmary; but the new spirit of social science has already almost forgotten that free medical relief was ever any disgrace to the recipient; it does not now carry with it disfranchisement or any of the other drawbacks which attach to the acceptance of outdoor relief. A new boundary line thus becomes a possibility which should be drawn not on social but on medical grounds.

### The Infirmaries' Weakness.

The great weak point of the Poor-Law infirmary is that the obscure case does not, as a rule, get the immense advantage of specialist diagnosis and treatment, while the valuable opportunities which such cases offer to the advancement of medical education are entirely lost. The waste of clinical material in Poor-Law infirmaries is incalculable; doctors and surgeons who have held appointments in them are aware that it is probably not far short in extent of that which is made use of in voluntary hospitals.

The whole burden of medical education has been thrown upon the voluntary hospitals, and, considering the limitations under which they work, they have performed the double task of meeting the needs of the sick poor and providing opportunities for medical education with admirable results. But the difficulty of finding sufficient clinical material increases every year, and the position now is that in Poor-Law infirmaries there are a number of cases which would be of great value in this respect, but which are never available; and in voluntary hospitals there are many cases of no educational value which occupy the beds for long periods.

Taking the whole body of applicants for free medical relief, there are enough deserving cases of an interesting kind to fill the beds of the voluntary hospitals of London, where they would have a far better chance of proper diagnosis and treatment than in the Poor-Law infirmary.

### A New Use for Infirmaries.

The infirmaries, since they offer no opportunities for medical education, should be used for the sick and injured, whose cases are of no special interest to the doctor or surgeon, the two classes corresponding to some extent to those in another position in life, who seek in the one case the specialist, in the other the general practitioner. It is an exchange of patients, therefore, which I suggest as a partial solution of some of the most difficult questions of the present position.

It is calculated that about a tenth of incomes from £300 to £700 a year is now paid away in rates and taxes; that is probably a conservative estimate, but, at any rate, it is sufficient to show that no scheme has any chance of acceptance which involves a heavy addition to the rates.

I have already suggested that before any close co-operation takes place between infirmaries and voluntary hospitals the taint of the workhouse must be removed, or we should find patients objecting in the strongest manner possible to being transferred from one to the other. There is already a body supported by the rates and covering the whole Metropolitan area which deals with infectious cases either from the Poor-Law or voluntary institutions.

The Metropolitan Asylums Board.

This is the Metropolitan Asylums Board. If the Poor-Law infirmaries were placed under the entire control of the Metropolitan Asylums Board and were re-named "hospitals," the mental connection with the workhouse would be destroyed, and the voluntary hospitals would gladly and easily work with an authority with which they have had business-like relationship for many years. Moreover, the efficiency of the infirmaries would be immensely increased by placing them under one uniform system of control. The Local Government Board has done much in recent years, but no amount of inspection can prevent the individual views of Guardians making themselves felt, often with disastrous results; while an organisation that depends mainly for success or failure upon the relieving officer stands self-condemned. The Metropolitan Asylums Board now controls successfully some 20,000 beds, and the authorities of voluntary hospitals would have confidence in handing patients over to their care.

A hospital desiring to transfer a case to an infirmary would take precisely the same steps that are now taken in respect to infectious cases; while, on the other hand, the medical superintendent of an infirmary could apply to a hospital for the transfer of any case which he considered of a sufficiently interesting nature to warrant such action.

On both sides there would be a strong inducement to carry on the arrangement, for the hospital authorities would be glad to empty a bed of a case which presented no features of interest; while the superintendent of an infirmary would know that he was reducing the general rate by sending a patient to a voluntary hospital.

Hospitals and Local Authorities.

The main point of friction between the infirmary and the hospital has been finance; the Poor-Law authorities look on the transfer of a case to them as an attempt to add to the local rates. This difficulty is caused by the fact that the hospital collects cases from a wide area, and wishes to transfer them to an infirmary which is intended to serve a much smaller district; and although the action of the Metropolitan Poor-Law Fund does, as a matter of fact, distribute a large part of the cost of indoor relief, a hospital cannot afford to irritate local authorities on whose consideration it is frequently dependent for an under-assessment to rates. But if all infirmaries were under the control of the Metropolitan Asylums Board, the transfer of a case would only affect the rates of the whole Metropolitan area; and since the Metropolitan Asylums Board would be theoretically responsible for the treatment of the sick poor in the whole of London, the fact that a case had first passed through the hands of a voluntary hospital could give no cause for complaint. On the other hand, the voluntary hospital would be relieving the rates of the burden of treating the serious and complicated cases which were transferred to its care.

An Exchange of Cases.

The voluntary hospitals are now doing all they can in treating the sick poor; the infirmaries do their share, and the combined accommodation of both is apparently sufficient. An exchange of cases between them could not, therefore, either add to the rates or throw an additional burden upon the voluntary hospital; it is merely a question of dividing differently the cases already treated by both agencies. Such an exchange would, however, throw open to medical education the vast number of cases now lost in Poor-Law infirmaries, and while thus tending to the advancement of knowledge and the better treatment of human suffering in future generations, it would give to these individuals an infinitely better chance of correct diagnosis, and therefore of recovery, than they have at present. Finally, it would relieve the Poor Law of the heavy responsibility of receiving and treating men and women who cannot get in Poor-Law institutions the skill which they should have in aiding them to battle against disease and death.

